# Enhanced Drainage Protocol in Large Amoebic Liver Abscess

**DOI:** 10.1055/s-0041-1740625

**Published:** 2021-12-23

**Authors:** Jignesh A. Gandhi, Pravin H. Shinde, Sadashiv N. Chaudhari, Amay M. Banker

**Affiliations:** 1 Department of Surgery, Global Hospital Mumbai, Parel, Mumbai, India; 2 Department of General Surgery, Seth G.S. Medical College & KEM Hospital, Mumbai, India

**Keywords:** amoebic liver abscess, drain, pigtail, biliary communication

## Abstract

**Background** Amebic liver abscess (ALA) contributes significantly to morbidity and mortality in patients of the developing world. Even though medical management is the primary modality of treatment, 15% of the cases are refractory and require intervention for drainage. Pigtail catheterization is inefficient and results in a long duration of hospital stay. So, we conducted a prospective observational study to determine the efficacy and safety of drainage of large ALA using a wide bore 24 French (Fr) drain compared with a conventionally used 10 Fr pigtail catheter.

**Materials and Methods** A single center prospective observational study was conducted over a period of 5 years and data of 122 patients was collected. After starting empirical medical therapy, patients underwent drainage of ALA with either a 10 French pigtail or a 24 Fr drain. The primary outcome variables were resolution of clinical symptoms such as fever and pain in abdomen, length of hospital stay, and resolution of abscess on imaging at day 3. Secondary outcome was complications related to the procedures.

**Results** Data of 122 patients was collected. Males constituted a vast majority (96%) of the study population and the fifth decade was the most common age group involved. Alcoholics had a higher chance of developing a large ALA. Sixty-eight patients underwent drainage of the ALA using a 24 Fr drain which resulted in faster resolution of symptoms (2.4 vs. 5.1 days, *p* -value 0.033), a shorter duration of catheter in situ (6.4 vs. 13.2, *p* -value 0.011), and a faster drainage of ALA (residual volume at day 3; 177 vs. 212 mL, *p* -value 0.021). Twenty-eight patients had a biliary communication of which 26 required therapeutic endoscopic retrograde cholangiopancreatography.

**Conclusion** In patients with a large ALA, placement of a wide bore 24 Fr catheter hastens recovery of the patients when compared with drainage with a standard 10 Fr pigtail catheter. Placement of a biliary stent serves as a useful adjunct for their management and it may obliviate the need for a major biliary diversion surgery.

Amoebiasis is a common parasitic infection affecting approximately 50 million people worldwide and resulting in approximately 100,000 deaths per year. 1 2 3 Ninety percent of such patients remain asymptomatic and the liver is affected in 3 to 9% of cases. This makes amebic liver abscess (ALA) the most common extraintestinal manifestation of amebiasis. 4 The mainstay of treatment for ALA is medical, but up to 15% of the cases may be refractory to medical management. 5 Presence of communication with the biliary tree is considered one of the reasons for non-resolution of ALA. 4 6 Imaging-guided percutaneous placement of a catheter has shown to be superior to needle aspiration for such refractory ALAs. 6 7 8 9 10 11 However, in our experience we found that the pigtail catheter becomes nonfunctional due to repeated blockage and requires repeated flushing which can introduce secondary infection ultimately resulting in a longer hospital stay. So, we conducted a prospective observational study to determine the efficacy and safety of drainage of large ALA using a wide bore 24 French (Fr) drain compared with a conventionally used 10 Fr pigtail catheter. We came across 28 cases of a biliary communication with the ALA in the past 5 years. In this study, we also present our experience in the management of such cases with the use of therapeutic endoscopic retrograde cholangiopancreatography (ERCP).

## Methodology

This was a single center prospective observational study conducted over a period of 5 years between October 2014 and June 2019 after approval from the institutional ethics committee. The amoebic etiology was suspected by the ultrasonographic (US) or computed tomographic (CT) appearance of the lesion and the diagnosis was confirmed by an enzyme-linked immunosorbent assay (ELISA). 12 Abscess cavities larger than 3 cm were considered large abscess for the purpose of this study. Patients with abscess cavities smaller than 3 cm in their greatest dimension; ruptured liver abscess; concomitant biliary tract malignancy; and uncorrectable coagulopathy were excluded from the study. A total of 122 patients were enrolled in the present cohort and were worked up in terms of detailed history and clinical examination. Laboratory and radiological investigations included a complete hemogram, liver function tests (LFTs), coagulation profile including prothrombin time and international normalized ratio, a US and a contrast enhanced CT scan of the abdomen.

After informed consent, all patients were started on intravenous metronidazole (500 mg every 8 hourly). Coagulopathy was corrected prior to any intervention. The modalities used along with medical therapy were insertion of a 10 Fr pigtail catheter and insertion of a 24 Fr drain with a blunt tipped trocar.

A 10 Fr pigtail catheter was used ALA showing an impending rupture on US (rim <10 mm), for left lobe abscess, and patients with abscess volume <200 mL and maximum diameter <4 cm on US. This was performed under USG guidance with all aseptic precautions.

A 24 Fr catheter insertion was performed for peripheral abscesses, abscesses with partially or uniquified ALA with a favorable anatomy on CT scan, and abscesses with maximum diameter >4 cm on US. Using a No.11 blade a small stab was made on the anaesthetized skin. Under US guidance, a 24 French drain with a blunt tipped trocar was then introduced into the abscess cavity, a technique similar to that described for trocar thoracostomy. 13

The aspirate was sent for routine microscopy and culture sensitivity and the antibiotics were adjusted accordingly. Patients with negative culture results were continued on the initial drug treatment for 14 days. Daily drain output was monitored for the presence of bilious contamination. Bile in drain was suggestive of a biliary fistula and those patients who had a consistent biliary output of >200 mL/d for >7 days were referred for an ERCP. After obtaining a cholangiogram, therapeutic ERCP was done which included a sphincterotomy followed by the placement of a 10-cm 7 Fr plastic pigtail stent up to the site of fistula wherever possible. Follow-up US was done on day 3 and all patients had a routine follow-up at 6 weeks.

The percutaneous drains were removed when drain output was <20 mL/d for 2 consecutive days or the maximum diameter of the abscess was <3 cm on follow-up US. Clinically stable patients with complete resolution of symptoms were discharged with pigtail catheter in situ with follow-up and removal on outpatient department (OPD) basis. Patients with a 24 Fr drain were discharged only after removal of drain. The biliary stent was removed after 6 weeks. The primary outcome variables were resolution of clinical symptoms such as fever and pain in abdomen, length of hospital stay, and resolution of abscess on imaging at day 3. Secondary outcome was complications related to the procedures. Chi-square test was used for the analysis of categorical variables and a logistic regression model was developed for continuous variables. Data was analyzed using SPSS 26.0 (Statistical Package for Social Sciences; IBM, Chicago, IL).

## Results

### Demographics

The present cohort consisted of 122 patients diagnosed with ALA. Males constituted a majority of the study population (96%). The average age of the cohort was 42 years. Ninety-three (76%) patients had a history of chronic alcohol consumption and diabetes (56%) was the most common comorbidity seen ( Table 1 ).

**Table 1:** Patient demographics

Total patients	122
Sex	
Male	117 (96%)
Female	5 (4%)
Age, years	42 (35–56)
Chronic alcohol use	93 (76.2%)
COMORBIDITIES a	
Diabetes mellitus	69 (56%)
Anemia	54 (44%)
Hypertension	48 (39%)
COPD	14 (11%)
Ischemic heart disease	1 (1%)

Abbreviation: COPD, chronic obstructive pulmonary disease.

Note: Values are listed as either number (percentage), mean (±standard deviation).

a Comorbidities coexisted in a few patients.

### Investigations

A total of 101 (82%) patients had leukocytosis on admission. Twenty-four (18%) patients had elevated bilirubin levels. Incidentally all these patients had a biliary communication with the liver abscess. Cultures were found to be positive in 18 of 122 (14.7%) of the cases. Among the pus culture positive cases *Escherichia coli* was isolated most frequently (8 of 18 culture positive patients).

**Fig. 1:**
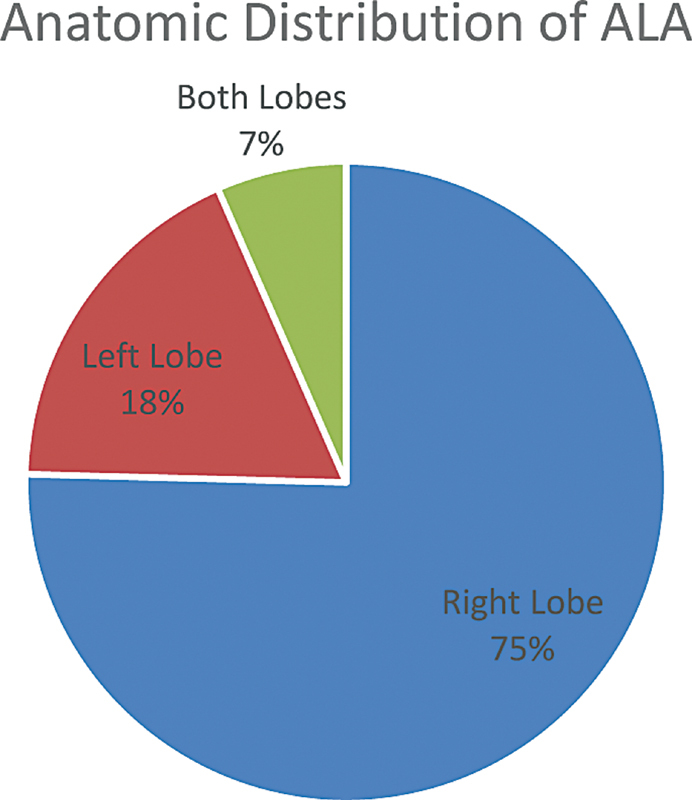
Anatomical distribution of liver abscess.

It was closely followed by *Klebsiella spp* . which was isolated in six cases. The rest were sterile abscesses ( Table 2 ). Out of the 122 patients, abscess was seen in the right lobe in 92 cases. Twenty-two cases were of left lobe abscesses while eight cases had abscesses involving both the lobes ( Fig. 1 ). The mean volume of the ALA was 656 mL (range 320–1,300 mL) on US corresponding to an average size of 4.2 cm (range 2.2–9.6 cm) in the largest diameter.

**Table 2:** investigations

Total patients	122
TLC counts/mm ^3^	18,2334 (±3,319)
Hemoglobin, mg/dL	11.5 (±2.14)
ALT, U/L	47.78 (±16.66)
AST, U/L	31.33 (±12.78)
ALP, U/L	196 (±76.98)
Total bilirubin, mg/dL	1.29 (±0.44)
US findings	
Size, cm	4.2 (±1.8)
Volume, mL	656 (±231)
Pus culture	
No growth	104 (85%)
*E. coli*	8 (6%)
*Klebsiella* species	6 (5%)
*Pseudomonas* species	2 (2%)
*Acinetobacter*	2 (2%)

Abbreviations: ALP, alkaline phosphatase; ALT, alanine transaminase; AST, aspartate transaminase; US, ultrasonography.

Note: Values are listed as mean (standard deviation) or number (percentage).

### Management

Sixty-eight (55.7%) patients underwent drainage with a 24 Fr catheter. The average volume of the ALA was 977 mL (622–1,300 cm). Mean residual volume on day 3 in this group was 177 mL with an average duration of catheter in situ being 6.4 days (range 3–22 days). Complete resolution of clinical symptoms was early (average 2.4 days). The mean length of hospital stay was 7.1 days (range 4–24 days) in this cohort. Twelve patients complained of pain at insertion site which was managed with oral analgesics. There was one case of hemorrhage within the ALA which was managed conservatively. One case of catheter migration required repositioning and there was one incidence of pneumothorax which was managed by intercostal tube insertion ( Table 3 ).

**Table 3:** Comparison of 24 Fr catheter vs. 10 Fr pigtail

	24 Fr catheter ( *n* = 68)	10 Frr pigtail ( *n* = 54)	*p* -Value
Age	41	44	0.988
Male sex	64 (94%)	53 (98%)	0.876
Volume, mL	977 (±344)	543 (±268)	0.046
Residual volume at day 3, mL	177 (±81)	212 (±94)	0.021
Duration of catheter, days	6.4 (±2.2)	13.2 (±4.2)	0.011
Resolution of symptoms, days	2.4 (±1.2)	5.1 (±3.7)	0.033
Length of hospital stay, days	7.1 (±3.7)	7.3 (±2.9)	0.512
Biliary communication	20 (29%)	8 (14%)	0.233
Complications			
Pain	12	4	–
Hemorrhage	1	0	–
Pneumothorax	1	0	–
Migration/removal	1	6	–
Block	0	14	–

Note: Continuous variables are listed as mean (standard deviation) and categorical variables as count (percentage).

Insertion of a 10 Fr pigtail was performed in 54 (44.3%) cases. The mean volume of the ALA was 543 mL in this group. Repeat US on day 3 showed a mean residual volume of 212 mL. The average duration of catheter in situ was longer (mean 13.2 days, range 6–24 days) and mean length of hospital stay was 7.3 days (4–18 days). The time taken for complete resolution of symptoms was 5.1 days. Accidental removal of pigtail was seen in six cases. A blocked pigtail was seen in 25% of the cases which required flushing.

### Statistical Analysis

Both the groups were homogenous in terms of age and sex distribution. In our study the average size and volume of the ALA in the 24 Fr catheter group was significantly higher (977 vs. 543 mL, *p* -value 0.046). Despite the larger average size of the abscess, there was a significantly faster resolution of symptoms seen in the larger 24 Fr drainage (2.4 vs. 5.1 days, *p* -value 0.033). The mean volume of the abscess on day 3 of drainage was also significantly lower in the group drained by the larger 24 Fr drain (177 vs. 212 mL, *p* -value 0.021). The average duration of drain in situ was also lower in the group drained by 24 Fr drain (6.4 vs. 13.2 days, *p* -value 0.011) ( Table 3 ). In three patients, small (2–3 cm) residual cavities were still present at 6 weeks after drainage; these patients were lost to further follow-up. There was no statistical difference between the two groups in terms of complete radiological resolution of the ALA.

### Biliary Communication

Biliary communication was noted in 28 cases. This subset of patients had a longer duration of hospital stay (mean 16.7 days) and they took longer for complete resolution of symptoms (mean 9.3 days). Elevated serum bilirubin levels (mean 4.3 mg/dL, range 2.2–9.9 mg/dL) and alkaline phosphatase levels (mean 344 IU/L, range 172–677 IU/L) were only seen in patients with a biliary communication. Amongst the patients with biliary communication, none had a left lobe abscess. The lesions with biliary communication tended to be larger with a mean volume of 990 mL (range 677–1,300 mL). Twenty of the 28 cases were those requiring a larger 24 Fr drain insertion. There was a spontaneous closure of the fistula in two cases. The remaining 26 cases were subjected to a therapeutic ERCP. No ERCP-related complications were noted in this cohort. Stenting led to the closure of biliary communication in all cases and no mortality was noted ( Table 4 ).

**Table 4:** Comparison of ala with and without biliary communication

	With biliary communication ( *N* = 28)	Without biliary communication ( *N* = 94)
Volume, mL	990 (±178)	491 (±228)
Serum bilirubin, mg/dL	4.3 (±1.1)	0.8 (±0.21)
Alkaline phosphatase, u/L	344 (±158)	167 (±49)
Duration of catheter, days	15.9 (±2.2)	8.6 (±4.2)
Resolution of symptoms, days	9.3 (±2.8)	4.1 (±1.7)
Length of hospital stay, days	16.7 (±3.6)	6.9 (±3.3)

Note: Continuous variables are listed as mean (standard deviation) and categorical variables as count (percentage).

## Discussion

Liver abscess is a major disease of the gastrointestinal system seen in the tropics. 14 ALA is a dead end for the trophozoite form of *Entamoeba histolytica* as there is no multiplication of the trophozoite form in the liver. The trophozoite survives on the hepatocytes leaving the connective tissue and the reticuloendothelial system intact. While metronidazole has remained the cornerstone of management of ALA since its acceptance, various studies have demonstrated early recovery with drainage of the abscess. 6 8 9 15 16 In the later stages ALA usually consists of acellular proteinaceous debris, and a brown fluid which is likened to “Anchovy Paste” consisting of necrotic hepatocytes. A 10 Fr pigtail catheter becomes nonfunctional due to blockage which may require repeated flushing which in turn can introduce secondary infections. This prompted us to drain the ALA with a wide bore 24 Fr catheter to achieve more efficient drainage. In a similar study by Gadahire and Shrotriya, they demonstrated earlier recovery of ALA when drained by a 20 Fr catheter compared with a 10 Fr pigtail. 9

The findings of our study demonstrate the superiority of a wide bore catheter drainage to conventional pigtail drainage in large ALA. Patients with large ALA who were drained with a 24 Fr catheter had faster resolution of symptoms and had a lower mean residual abscess volume on day 3. These findings are similar to those reported by Kumar and Singh. 17 The wide bore of the catheter allowed for a faster drainage of the abscess which explains earlier recovery in our patients. While earlier studies have shown a decrease in the length of hospital stay, no statistical difference was found in the present study. 9 17 In both these series, patients were discharged with the catheter in situ while no patient in the present study was discharged with the 24 Fr catheter in situ in this study.

Complications were noted in 25% of the cases of the catheter group with pain at the site of insertion being the most common (17%). All these cases were managed with analgesics. A pneumothorax was noted in one case which was managed by the insertion of the thoracic tube. The patient had an uneventful recovery and was discharged on postoperative day 16. One patient developed hemorrhage into the abscess cavity which was managed conservatively. While pain was less in the pigtail group, accidental removal of the pigtail was seen in six patients. All these patients required a repeat intervention. Twenty-five percent of the patients complained of a blocked pigtail requiring repeated flushing and resulting in a longer mean duration of pigtail in situ.

ALA is found to be 3 to 10 times more common in males. 18 In this study, the males constituted 96% of the study population. The fifth decade was the most affected age group. The clinical presentation of our patients was similar to that described in previous reports. 11 18 19 Seventy-six of the patients had a history of chronic alcohol use in the present study. Duration of alcohol use has been shown to be a risk factor for the development of ALA. 20 It was seen in animal studies that increased iron deposition increases the invasiveness of *E. histolytica* . Makkar et al hypothesized that since chronic alcoholism increases iron deposition in liver, it would explain the increased risk of ALA in chronic alcoholics. 21 A majority of our patients also presented with leukocytosis. Presentation with jaundice proved to be a useful clinical marker of abscess erosion into the biliary tract. Varying incidence (23–27%) of jaundice has been reported in patients with ALA. 6 22 23 While many pathogenetic mechanisms have been suggested, obstruction of the biliary system by the abscess is most widely accepted. 6 24 Nigam et al showed that multiple, large abscesses, have been directly related to the elevation of serum bilirubin levels. 23 Another possible mechanism is that large ALA compresses the biliary radicles producing jaundice and finally penetrates the tough fibrous vasculobiliary sheath which surrounds the portal triad structures with resultant biliary communication. 6 All our patients with jaundice showed a rapid decline in serum bilirubin to normal levels after drainage of the ALA, again establishing obstruction as a cause of jaundice in these patients. Pus cultures were negative in 85% of the cases and *Escherichia coli* and *Klebsiella* were the most commonly isolated organisms. This result is similar to the published literature. 11 25 26

The incidence of biliary communication was 22% in the present study which is similar to that reported in existing literature. 4 6 16 The presence of biliary communication was associated with the need of a longer duration of hospital stay. These patients also had a larger abscess, were more frequently associated with jaundice, and required catheter drainage for a longer duration. Agarwal et al also presented findings similar to these. 6 In two patients there was a spontaneous closure of the biliary fistula probably due to the tamponading effect of the hepatic parenchyma and low pressure of the now unobstructed biliary system after drainage of the ALA as suggested by Do et al. 27 The remaining cases required a therapeutic ERCP with sphincterotomy and placement of a stent for resolution. There was an uneventful recovery in all these cases and no patient required a surgical biliary diversion.

### Limitations

Our study is limited by the fact that it is a single institution observational study with no randomization. This may introduce a selection bias and further randomized controlled prospective studies are recommended. Some of the data in this study was non-normally distributed and a larger sample size is necessary to confirm our findings.

## Conclusion

In patients with a large ALA, placement of a wide bore 24 Fr catheter may be considered as an alternative to the established 10 Fr pigtail drainage to hasten recovery of the patients. Biliary communication should be suspected in patients of ALA presenting with jaundice and placement of a biliary stent serves as a useful adjunct for their management and it may obliviate the need for a major biliary diversion surgery.
